# Education and pandemic SARS-CoV-2 infections in the German working population – the mediating role of working from home

**DOI:** 10.5271/sjweh.4144

**Published:** 2024-04-01

**Authors:** Benjamin Wachtler, Florian Beese, Ibrahim Demirer, Sebastian Haller, Timo-Kolja Pförtner, Morten Wahrendorf, Markus M Grabka, Jens Hoebel

**Affiliations:** 1 Department of Epidemiology and Health Monitoring, Robert Koch Institute, Berlin, Germany.; 2 Institute for Medical Sociology, Health Services Research and Rehabilitation Science, University of Cologne, Cologne, Germany.; 3 Department of Infectious Disease Epidemiology, Robert Koch Institute, Berlin, Germany.; 4 Research Methods Division, Faculty of Human Sciences, University of Cologne, Cologne, Germany.; 5 Institute of Medical Sociology, Centre for Health and Society, Medical Faculty, Heinrich-Heine-University of Dusseldorf, Dusseldorf, Germany.; 6 Socio-economic Panel, German Institute for Economic Research, Berlin, Germany.

**Keywords:** COVID-19, infection risk, mediation analysis, socioeconomic inequalities, work from home

## Abstract

**Objectives:**

SARS-CoV-2 infections were unequally distributed during the pandemic, with those in disadvantaged socioeconomic positions being at higher risk. Little is known about the underlying mechanism of this association. This study assessed to what extent educational differences in SARS-CoV-2 infections were mediated by working from home.

**Methods:**

We used data of the German working population derived from the seroepidemiological study “Corona Monitoring Nationwide – Wave 2 (RKI-SOEP-2)” (N=6826). Infections were assessed by seropositivity against SARS-CoV-2 antigens and self-reports of previous PCR-confirmed infections from the beginning of the pandemic until study participation (November 2021 – February 2022). The frequency of working from home was assessed between May 2021 and January 2022.We used the Karlson-Holm-Breen (KHB) method to decompose the effect of education on SARS-CoV-2 infections.

**Results:**

Individuals with lower educational attainment had a higher risk for SARS-CoV-2 infection (adjusted prevalence ratio of low versus very high = 1.76, 95% confidence interval 1.08–2.88; P=0.023). Depending on the level of education, between 27% (high education) and 58% (low education) of the differences in infection were mediated by the frequency of working from home.

**Conclusions:**

Working from home could prevent SARS-CoV-2 infections and contribute to the explanation of socioeconomic inequalities in infection risks. Wherever possible, additional capacities to work remotely, particularly for occupations that require lower educational attainment, should be considered as an important measure of pandemic preparedness. Limitations of this study are the observational cross-sectional design and that the temporal order between infection and working from home remained unclear.

The outbreak of the newly emerged severe acute respiratory syndrome coronavirus type 2 (SARS-CoV-2) in the early days of 2020, later declared a pandemic by the World Health Organization (WHO) on 11 March 2020, has challenged societies worldwide on a historic scale. Since the beginning of the outbreak, hundreds of millions of individuals have been infected worldwide and almost 7 million deaths due to the new coronavirus disease 2019 (COVID-19) were reported until the WHO declared the end of the global health emergency on 5 March 2023 (1). Already during the early phases of the COVID-19 pandemic, evidence emerged that people in a lower socioeconomic position (SEP) might have been infected more frequently with SARS-CoV-2 than people in higher SEP (2). Today, there is plenty of evidence from numerous studies worldwide demonstrating that the SARS-CoV-2 infection risk was unequally distributed across societies during the pandemic, mostly to the disadvantage of those with lower SEP (3–5).

But how these socioeconomic inequalities in SARS-CoV-2 infections exactly arose is still not fully understood. There are different synopses available that differentiate possible pathways through which socioeconomic inequalities in SARS-CoV-2 infections might be explained, but empirical evidence remains limited so far. For instance, Clare Bambra described four main interconnected pathways (unequal exposure, unequal transmission, unequal susceptibility and unequal treatment) whereby existing social inequalities shape the spread and distribution of newly emerged infectious diseases (6). For some of these pathways, empirical evidence exists but still remains inconclusive. One major driver of inequalities in SARS-CoV-2 infections that is mentioned frequently is the unequal exposure as a result of unequal working conditions (6, 7).

It has been described that some occupations, such as healthcare workers (8–10), indoor trade and process and plant workers (11, 12) had higher risks of infection during the early phases of the pandemic. Beale et al. found that these differences were partly due to differences in work-related close contacts (11) and Reuter et al. found that the SARS-CoV-2 infection risk was generally twice as high for essential workers than for non-essential workers (9). Attendance at the workplace during the COVID-19 pandemic seems generally to be a risk factor for a SARS-CoV-2 infection (13, 14). In line with these findings, some studies found that the possibility to work from home was associated with a lower infection risk (15–18). However, the evidence is inconsistent here, as some studies found no associations between the possibility to work from home and SARS-CoV-2 infection risk (19, 20) or even associations to the disadvantage of those who were working remotely (21). The employees’ position in the occupational system is strongly shaped by their educational attainment, and studies have shown that highly qualified employees generally have more opportunities to work from home, which was especially evident during the COVID-19 pandemic (22, 23).

In order to slow down the spread of the virus, access restrictions to the workplace were in place in multiple countries and working from home was often recommended, at least during some periods of the pandemic. Some countries, including Germany, implemented legislation that made it mandatory for employers to offer the possibility to work from home whenever working remotely was regarded as feasible. But in the case of Germany, this legislation was only effective between January and June 2021 (24). However, offering the possibility to work from home appears to be an interesting potential intervention to prevent infection, with limited negative effects on the wider economy.

But the possibility to work from home is unequally distributed within the workforce (25, 26). Individuals with higher educational attainments more frequently tend to have the possibility to work remotely than socially more disadvantaged individuals (22, 23) therefore the recommendations or orders to work from home might have discriminated individuals in lower SEP and might at least partly explain the observed socioeconomic inequalities in SARS-CoV-2 infection risk. To our best knowledge, no study has examined and quantified how much of these inequalities can be explained by inequalities in the possibility to work from home. This knowledge is needed in order to evaluate the recommendation to work remotely whenever possible and to identify those who are not yet able to work from home and increase the efforts to make working from home more available to those who could not yet benefit from these recommendations.

We therefore conducted this study in order to address the following research questions: Was the association between educational attainment as a measure of SEP and pandemic SARS-CoV-2 infection risk in the working population mediated by the possibility to work from home? How much of the association between educational attainment and pandemic SARS-CoV-2 infection risk was mediated by the unequal possibility to work from home?

## Methods

### Study design and population

We used data from the German population-based seroepidemiological study “Corona Monitoring Nationwide – Wave 2 (RKI-SOEP-2)” and the Socio-Economic Panel (SOEP) study. The RKI-SOEP-2 study was based on the SOEP, a dynamic cohort based on nationwide random samples of individuals living in private households across Germany. All individuals who participated in the SOEP survey wave in 2021 and their household members aged ≥14 years were invited to participate in the RKI-SOEP-2 study. Data collection took place between November 2021 and February 2022. A questionnaire and test-kit for self-sampling capillary blood to detect immunoglobulin G (IgG) antibodies against the spike protein (anti-S-antigen) and the nucleocapsid protein of SARS-CoV-2 (anti-N-antigen) was sent to each target person. In order to achieve a high participation rate, respondents were informed that they would receive the results of their blood test and a monetary incentive (€10 for adults and €5 for adolescents) if all the documents were completed and returned. The questionnaire covered topics such as previously experienced SARS-CoV-2 infection, COVID-19 vaccination status, the willingness to be vaccinated and questions on general health status and health behaviors. The Ethics Committee of the Berlin Chamber of Physicians approved the study (Eth-33/20) in compliance with the Declaration of Helsinki. A detailed study description, including detailed information on response rates and non-response analyses, can be found elsewhere (27, 28).

### Outcome: SARS-CoV-2 infections

SARS-CoV-2 infection status was assessed by using individual self-reports and serological assays for SARS-CoV-2 antibodies (29). The Euroimmun enzyme-linked immunosorbent assays (ELISA) anti-SARS-CoV-2-QuantiVac and anti-SARS-CoV-2-NCP were used for the detection of anti-S (S1 domain of the spike protein) and anti-N (nucleocapsid protein, NCP) antibodies. To determine seropositivity for anti-N antibodies in dried blood samples, the ratio cut point for serum samples provided by the manufacturer was adapted from 1.1 to 0.95 (30). A positive SARS-CoV-2 infection status was defined as either having self-reported a previous PCR-confirmed infection or seropositivity for anti-N antibodies or, in the absence of self-reported vaccination, seropositivity for anti-S antibodies (29). Infections might have occurred between the onset of the pandemic in Germany and the date of individual study participation.

### Exposure: educational attainment

Self-reported educational attainment from the SOEP wave in 2020 (or the latest available data from earlier waves) was used. Education was classified with the 2011 version of the International Standard Classification of Education (ISCED) (31). Participants’ highest school and vocational qualifications were classified as low (lower secondary education or below, ISCED levels 0–2), medium (upper secondary or post-secondary education, ISCED levels 3 and 4), high (lower tertiary education: Bachelor’s degree or equivalent, ISCED levels 5 and 6) and very high (upper tertiary education: Master’s degree or equivalent or doctorate ISCED levels 7 and 8).

### Mediator: working from home

The frequency of working from home was assessed in SOEP core wave 2021 (data collection between May 2021 and January 2022). Participants were asked how often they were working from home. We coded the variable into four categories (i): not working from home (ii); every two weeks or less frequently (iii); several times a week; and (iv) daily. For sensitivity analyses, the variable was also dichotomized into working from home and not working from home.

### Confounding variables

A directed acyclic graph (32) was drawn to identify a minimal sufficient adjustment set to estimate the total effect of education on infection risk and the mediation of this association by the frequency of working from home (supplementary material, URL, 1 and 2). Age, sex, migration experience, federal state and urban versus rural residence [based on characteristics of the district of residence according to official statistics (33)] were identified as the minimal sufficient adjustment set using DAGitty version 3.1 (34). Household composition is known to be a predictor of SARS-CoV-2 infection but was regarded as a potentially mediating variable between educational attainment and SARS-CoV-2 infection risk here and was therefore not included into the minimal sufficient adjustment set. A sensitivity analysis was conducted by including household composition (“single household”, “multiple persons without children under 16” and “multiple persons with children under 16”) into the adjustment set of the analysis.

### Statistical analysis

Descriptive results are presented as proportions and prevalences with 95% confidence intervals (95% CI). In order to establish mediation, the following conditions must hold according to the traditional approach by Baron & Kenny (35): (i) the exposure variable (education) must be significantly associated with the mediator (frequency of working from home); (ii) exposure (education) must be significantly associated with the outcome (SARS-CoV-2 infection); and (iii) the mediator (frequency of working from home) must be significantly associated with the outcome (SARS-CoV-2 infection). If all these conditions hold in the predicted direction, the association of the exposure variable on the outcome must become weaker when the mediator variable is added to the regression model. In addition, significant exposure–mediator interaction must be ruled out.

The association between educational attainment and risk of SARS-CoV-2 infection, as well as between frequency of working from home and risk of SARS-CoV-2 infection, was estimated by calculating prevalence ratios (PR) with 95% CI and P-values using Poisson regressions with household-clustered standard errors. The models were adjusted for age, sex, migration experience, federal state and urban versus rural residence. In the next step, a third model that included both the exposure and mediator variables was calculated using the same adjustment set. An additional regression model with an interaction term between education and frequency of working from home was calculated to assess the exposure–mediator interaction while adjusting for the minimal sufficient adjustment set.

We conducted a decomposition analysis of the effect of education on SARS-CoV-2 infection risk by using the Karlson-Breen-Holm (KHB) method (36, 37). The KHB method allows to recover the degree to which a variable mediates the relationship between the exposure and the outcome and is unaffected by the rescaling or attenuation bias that might arise in simple cross-model comparisons in non-linear models (38). The method was implemented by using the Stata khb package and household-clustered robust standard errors were calculated (38).

The analyses were calculated using weighting factors that compensated for systematic panel attrition, systematic non-response and adjusted the sample to match the official German population statistics by age, sex, citizenship (German versus non-German), federal state, household type and size, as well as owner-occupied housing (27, 28). Details of the weighting procedures and non-response can be found elsewhere (28). The sample population was restricted to the working population aged 18–67 years. All analyses were conducted using the survey data functionality of Stata version 17.0 (StataCorp LLC 2021, College Station, TX, USA). The reporting of this study follows the Strengthening the Reporting of Observational Studies in Epidemiology (STROBE) guidelines (39).

## Results

A total of 11 162 individuals from 6760 households took part in the RKI-SOEP-2 study between November 2021 and February 2022. A majority of 74% of the sample population participated in November and December 2021. The overall response rate was 53.7%. We included 6826 individuals into this study who were 18–67 years old and described themselves as “working”. The description of the sample population according to the SARS-CoV-2 infection status is shown in table 1.

**Table 1:** Description of the sample population by SARS-CoV-2 infection status.

		Total ^a^			No SARS-CoV-2 infection			SARS-CoV-2 infection ^b^	
		N ^c^	% ^d^		N ^c^	% ^d^		N ^c^	% ^d^
		6826	100		6018	89		805	11
Sex									
	Female	3171	53.6		3214	51.5		365	43.9
	Male	3655	46.5		2804	48.5		440	56.1
Age (years)									
	18–34	1466	30.2		1284	30.4		181	22.5
	35–49	2276	32.1		1964	30.6		311	38.6
	49–67	3084	37.7		2770	39		313	38.9
Education									
	Very high	1170	14.8		1074	15.4		96	9.9
	High	1667	23.1		1472	23		195	23.8
	Medium	3092	49.2		2718	48.9		373	51
	Low	534	8.3		445	8		88	10.2
	Missing	363	4.7		309	4.6		53	5.0
Possibility to work from home									
	No possibility	3282	51.3		2852	50.3		429	58.5
	Every two weeks or less frequently	700	10		616	10		84	9.1
	Several times per week	1200	16.4		1096	17		104	11.1
	Daily	967	15.2		898	15.9		69	9.5
	Missing	677	7.3		556	6.8		119	11.8
Migration experience									
	Yes	819	82.2		670	16.3		148	26
	No	5959	17.4		5304	83.3		653	74
	Missing	48	4		44	0.4		4	0.3
Residential area									
	Urban	4545	69.4		4046	70		496	64.7
	Rural	2204	29.3		1906	28.9		298	32.9
	Missing	77	1.3		66	1.1		11	2.3

^a^ The numbers in the table do not fully add up to the totals of n = 6,826 because the infection status values for three participants could not be retrieved. ^b^ Self-reported previous PCR-confirmed infection or seropositivity for anti-N antibodies or, in the absence of self-reported vaccination, seropositivity for anti-S antibodies against SARS-CoV-2. ^c^ Unweighted number of participants. ^d^ Weighted proportions.

A total of 805 individuals or 11% of the sample population had a SARS-CoV-2 infection and the proportion of SARS-CoV-2-infected individuals was higher among those with lower formal education. While 13.6% (95% CI 9.4–19.4) in the low educated group had a previous SARS-CoV-2 infection, only 7.4% (95% CI 5.2–10.4) in the very highly educated group had a previous SARS-CoV-2 infection. Furthermore, a larger proportion of the highly educated group reported working more frequently from home than those with a lower formal education (table 2). The exposure (educational attainment) was associated with the mediator (possibility to work from home): those with a very high education had a 9.45-fold (95% CI 5.49–16.28; P<0.0001) higher probability to work from home several times per week in comparison to those with low educational attainment after adjustment for age, sex, migration experience, federal state and urban versus rural residence. Educational attainment (exposure) and the possibility to work from home (mediator) were both associated with the SARS-CoV-2 infection status (outcome), as shown in table 3. The prevalence of a SARS-CoV-2 infection was 1.76 times higher in the group with low education in comparison to those in the group with very high education (95% CI 1.08–2.88; P=0.023) after adjusting for age, sex, migration experience, federal state and urban versus rural residence. When the frequency of working from home was added to the model, the total effect of education on infection risk was diminished to a prevalence ratio of 1.27 (95% CI 0.78–2.09; P=0.338) between the groups with low and very high educational attainment (table 3). We found no significant exposure–mediator interaction (supplementary material 3).

**Table 2:** Possibility to work from home by educational attainment. [CI=confidence interval]

		No possibility			≤ Every two weeks			Several times per week			Daily			Missing	
		N ^a^ (%) ^b^	95% CI		N ^a^ (%) ^b^	95% CI		N ^a^ (%) ^b^	95% CI		N ^a^ (%) ^b^	95% CI		N ^a^ (%) ^b^	95% CI
Total		3282 (51.3)	49.4–53.1		700 (9.9)	8.9–11		1200 (16.4)	15.1–17.7		967 (15.2)	13.9–16.6		677 (7.3)	6.5–8.3
Education	Very high	225 (17.2)	14.2–20.7		151 (12.2)	9.8–15.2		380 (33.5)	29.5–37.8		342 (32.6)	28.5–37		72 (4.5)	2.9–6.8
	High	633 (39.4)	35.8–43.1		234 (13.6)	11.3–16.2		419 (24.7)	21.8–27.9		271 (17)	14.4–20.0		110 (5.3)	3.9–7.2
	Medium	1950 (64.2)	61.6–66.6		258 (8.6)	7.3–10.2		338 (10)	8.5–11.6		301 (11.2)	9.7–13.1		245 (6)	5.0–7.3
	Low	335 (71.3)	65.3–76.6		27 (4.2)	2.3–7.5		14 (2.3)	1.1–4.7		13 (3.4)	1.5–7.3		145 (18.9)	14.4–24.3
	Missing	139 (46.8)	38.1–55.7		30 (7.7)	4.4–13.1		49 (12.7)	8.1–19.2		40 (13.2)	8.3–20.2		105 (19.7)	14.3–26.5

^a^ Unweighted totals. ^b^ Weighted percentages.

**Table 3:** Relative risk of SARS-CoV-2 infection by educational attainment and frequency of working from home. [PR=prevalence ratio; CI = confidence interval; ref.=reference category].

		Model 1 ^a^				Model 2 ^a^				Model 3 ^a^		
		PR	95% CI	P-value		PR	95% CI	P-value		PR	95% CI	P-value
Education												
	Very high (ref.)	(ref.)	(ref.)	(ref.)						(ref.)	(ref.)	(ref.)
	High	1.51	1.01–2.26	0.043						1.34	0.89–2.02	0.16
	Medium	1.66	1.15–2.39	0.007						1.34	0.92–1.96	0.126
	Low	1.76	1.08–2.88	0.023						1.27	0.78–2.09	0.338
Possibility to work from home												
	No possibility					(ref.)	(ref.)	(ref.)		(ref.)	(ref.)	(ref.)
	≤ Every two weeks					0.85	0.60–1.19	0.337		0.88	0.63–1.22	0.436
	Several times per week					0.61	0.43–0.86	0.005		0.65	0.45–0.92	0.017
	Daily					0.55	0.39–0.79	0.001		0.59	0.41–0.86	0.006

^a^ Models 1–3 = Poisson regression models adjusted for age, sex, migration experience, federal state and urban versus rural residence.

The results of the decomposition analysis according to the KHB method are shown in table 4. The association of education with SARS-CoV-2 infection risk was mediated by the frequency of working from home. The proportion of the total effect that was mediated by the frequency of working from home varied between the education groups: 27% of the effect of education on SARS-CoV-2 infection risk was mediated by the frequency of working from home in the highly educated group, while it was 42% in the group with medium and 58% in the group with low education (table 4).The sensitivity analysis, which included the household composition into the adjustment set of the analyses showed that the results varied only marginally (supplementary material 4).

**Table 4:** Decomposition of the total effect of education on SARS-CoV-2 infection risk according to the Karlson-Holm-Breen (KHB) method. [SE=standard error; CI=confidence interval; ref.=reference category.

Education	Effect	Model 1 ^a^					Model 2 ^b^			
		Coefficient (SE)	95% CI	P-value	Mediation % ^c^		Coefficient (SE)	95% CI	P-value	Mediation % ^c^
Very High	(ref.)	(ref.)	(ref.)	(ref.)	(ref.)		(ref.)	(ref.)	(ref.)	(ref.)
High	Total	0.54 (0,23)	0.09–0.99	0.018			0.47 (0.23)	0.02–0.92	0.041	
	Direct	0.39 (0.23)	-0.07–0.85	0.096	28.1		0.34 (0.24)	-0.11–0.80	0.146	26.8
	Indirect	0.15 (0.06)	0.16–49.57	0.011			0.13 (0.06)	0.02–0.23	0.024	
Medium	Total	0.60 (0.21)	0.20–1.01	0.004			0.58 (0.21)	0.17–0.99	0.005	
	Direct	0.31 (0.22)	-0.11–0.73	0.149	48.5		0.34 (0.22)	0.09–0.75	0.119	42
	Indirect	0.29 (0.09)	0.12–0.46	0.001			0.24 (0.08)	0.08–0.40	0.003	
Low	Total	0.80 (0.29)	0.23–1.36	0.006			0.67 (0.28)	0.11–1.22	0.020	
	Direct	0.34 (0.30)	-0.24–0.92	0.256	57.7		0.28 (0.29)	-0.30–0.86	0.343	58.1
	Indirect	0.46 (0.11)	0.24–0.68	<0.0001			0.39 (0.11)	0.18–0.60	<0.0001	

^a^ Model 1 = adjusted for age and sex ^b^ Model 2 = adjusted for age, sex, migration experience, federal state and urban versus rural residence ^c^ Mediation % = (Indirect effect / Total effect) × 100

## Discussion

This is the first study to examine the mediating effect of working from home during the COVID-19 pandemic on educational inequalities in pandemic SARS-CoV-2 infection among Germany’s working population. We found that individuals with lower educational attainment had a higher risk for SARS-CoV-2 infection during the pandemic than those with higher education. At the same time, individuals with lower educational attainment were less frequently able to work from home and those with fewer possibilities to work from home had higher risks for infection with SARS-CoV-2. Our decomposition analysis showed that a substantial proportion of the educational inequality in SARS-CoV-2 infection risk was mediated by differences in the frequency of working from home between educational groups, with the highest contribution to explanation of the differences being among those with low formal education.

Our results are in line with previous findings that showed an association between the possibility to work from home and SARS-CoV-2 infection risk. The majority of these studies showed that those who were not able to work remotely had a higher risk of infection during the pandemic (15–18, 40–46). In contrast, one study among employees of a big Swiss company found that those who worked from home most of the time during the year 2020 had higher odds for a SARS-CoV-2 infection in comparison to colleagues who only sometimes or never worked from home (21). However, these results were not statistically significant, the sample was very small and the study design might have been particularly prone to selection bias. In contrast to our results, another study from Cologne, Germany, found no statistically significant association between working from home and SARS-CoV-2 infection risk after adjusting for multiple other variables (47). However, the sample was relatively small and restricted to the population of just one German city. Our results are also in line with most of the available evidence on socioeconomic inequalities in SARS-CoV-2 infection risk that showed higher SARS-CoV-2 infection risks for individuals in lower SEP. By using the level of educational attainment as a measure of SEP, our study adds to the previously existing evidence that a substantial part of the socioeconomic inequalities in pandemic SARS-CoV-2 infection risk can be explained by the frequency of working from home.

A literature search in “LitCovid”, a literature hub for tracking scientific research papers on COVID-19 provided by the US National Institute of Health (48), revealed that no study investigating the mediating effect of working from home on the association between formal education and SARS-CoV-2 infection risk was published in the PubMed database until 1 August 2023. We therefore assume that this is the first study – not only in Germany but internationally – to assess the role of working from home in explaining these inequalities. By using data from a population-based seroepidemiological study, we were able to include both previously known and unknown infections with SARS-CoV-2. Another strength of this study is the high external validity to the German working population living in private households. We used a directed acyclic graph to systematically identify potentially confounding variables and adjusted our analyses to the minimal sufficient adjustment set. The KHB method was used to minimize the rescaling problem that arises when comparing regression coefficients between same-sample nested models. We tested for exposure–mediator interactions and found no significant interaction. Furthermore, we did not see a strong correlation between the error terms of the mediated and non-mediated model, which points towards the absence of relevant residual confounding in our analysis (49).

Despite these strengths, our study has some limitations that should be kept in mind when interpreting the results. Firstly, our data came from a cross-sectional observational study, which may to a certain degree have been affected by selection bias. The detailed analyses of the reasons for non-response showed that among other factors, lower educational attainment was associated with higher levels of non-response (28). This might have led to an underestimation of educational inequalities in SARS-CoV-2 infection risk despite the fact that we used weights to correct our analyses for systematic non-response. In addition, the information on the frequency of working from home came from the SOEP survey wave in 2021 (study period May 2021 to January 2022), whereas our outcome was assessed in the RKI-SOEP-2 study (study period November 2021 to February 2022). Infections might have occurred prior to the assessment of the frequency of working from home as infections were partly assessed retrospectively when participants were asked about previous PCR confirmed infections. This and the observational design of our study means that causal interpretations of our results are impossible. The possibility to work remotely might have changed between the two timepoints, leading to the possibility of some misclassification and the knowledge of a previous infection might have influenced the recollection of the possibility to work from home. Additionally, we do not know exactly when the SARS-CoV-2 infections occurred and were not able to differentiate between first infections and subsequent re-infections. As we know that individuals with lower educational attainment have a higher SARS-CoV-2 infection risk, we might have missed re-infections that are more probable in individuals with low education. This may have led to an underestimation of educational inequalities and the protective effect of working from home by not counting these additional subsequent infections. In this study, we have focused on the mediating effect of working from home, but educational inequalities might also arise through other mechanisms that were not assessed in this study. It cannot be ruled out that some of these unobserved alternative explanations, such as differences in protective behavior, inequalities in housing conditions and on the neighborhood level, etc., might also have influenced our findings. We conducted a sensitivity analysis and additionally included the household composition as potentially confounding variable and found that our results did not change. Another limitation of our study is, that while we used the most appropriate data source available with a high number of participants, we were still not able to stratify our analyses for gender while simultaneously stratifying by education and sufficiently adjusting for potentially confounding variables. As the gender distribution differs across different occupations, and the infection risk within occupations may differ by gender (50), further gender-specific analyses are desirable.

### Concluding remarks

We analyzed, for the first time, what effect working from home has in the explanation of educational inequalities in SARS-CoV-2 infection risk and found that working from home explained 27–58% of these differences. However, limitations of this study were the cross-sectional design and the impossibility to establish a clear temporal order between the mediator and the outcome that make a causal interpretation impossible. The results of our study suggest that enabling as many individuals as possible to work from home during outbreaks of acute respiratory diseases might be promising for public health, pandemic preparedness planning and politics because it could reduce overall infections and possibly also socioeconomic inequalities in infection risk. It should particularly be considered if more jobs that require lower levels of education could be done remotely in order to reduce health inequalities during epidemics. In addition, those who are not able to work from home should be prioritized during the distribution of effective protective equipment and preventive interventions such as vaccination campaigns. Those industries where remote working is not possible should have effective infection prevention and control plans in place. Enabling a growing proportion of the population to work from home should further be planned and realized not only during but particularly between infectious disease epidemics in order to be prepared for the next outbreak. The further health consequences of working from home are the recent focus of many international studies and the effect of working from home on both the individual’s mental and physical health and productivity is still at the center of an ongoing debate (51–53). Working from home might, on the one hand, be associated with negative side effects such as higher levels of musculoskeletal pain (54), depression or alcohol consumption (55, 56), less physical activity, etc (57). On the other hand, working from home might lead to a reduction of contacts with infected individuals, longer sleeping times, more time for physical activity, an enhanced possibility to arrange working and personal life and better productivity (58, 59). Further research with a health equity focus is needed to fully understand the effect of working from home on individuals’ health and wellbeing. A better understanding of the various effects of working from home could help to improve the work environment at home and reduce potential adverse health impacts if it becomes necessary again to limit human mobility and physical contacts on a large scale during the next epidemic.

### Ethics approval and consent to participate

The Ethics Committee of the Berlin Chamber of Physicians approved this study (Eth-33/20) in compliance with the Declaration of Helsinki. Informed consent to participate was obtained from all individual participants included in the study.

## Supplementary material

Supplementary material

## Data Availability

The data cannot be made publicly available because informed consent from participants did not cover the public deposition of data. However, the data underlying the analysis in this article is archived in the SOEP Research Data Centre in Berlin and can be accessed on site upon reasonable request (www.diw.de/en/diw_01.c.601584.en/data_access.html).
