# Supplementary material for: Education and pandemic SARS-CoV-2 infections in the German working population – the mediating role of working from home
Source: Scand J Work Environ Health. 2024 Mar 27;50(3):168–77. doi: 10.5271/sjweh.4144 (PMC11064849; doi:10.5271/sjweh.4144)
Supplement: Supplementary material [file SJWEH-50-168-S001.pdf]

Education and pandemic SARS-CoV-2 infections in the German working population – the mediating role of working from home[1]

by Benjamin Wachtler, MD, MSc,[1] Florian Beese, MPH,[1] Ibrahim Demirer, PhD, MSc,[2] Sebastian Haller, MD, MPH, MSc,[3] Timo-Kolja Pförtner, PhD, MSc,[4] Morten Wahrendorf, PhD, MSc,[5] Markus M Grabka, DrPH, MA,[6] Jens Hoebel, DrPH, MSc [1]

1. Supplementary material

2. Correspondence to: Dr Benjamin Wachtler, ORCID ID 0000-0002-3959-5676, Department of Epidemiology and Health Monitoring, Robert Koch Institute, Nordufer 20, 13353 Berlin, Germany. [E-mail: wachtlerb@rki.de]

Supplementary Material 1 Simplified directed acyclic graph (DAG) for the mediation analysis

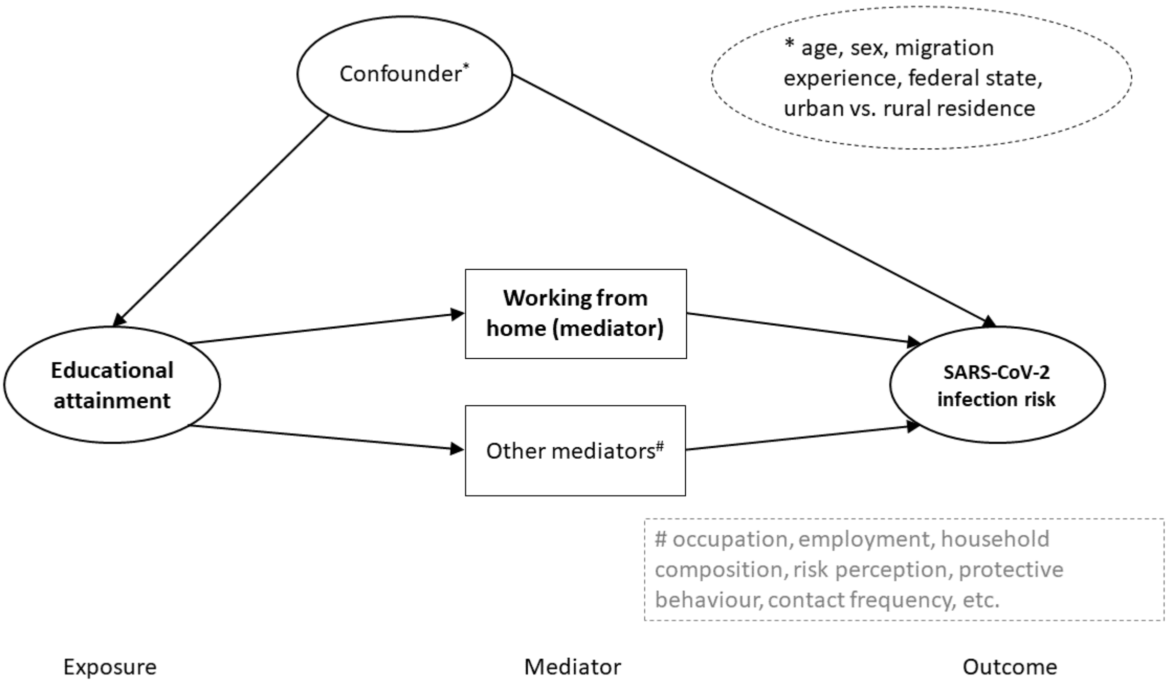

Supplementary Material 2 Model code for the comprehensive directed acyclic graph (DAG) for the mediation analysis for the use with the R package dagitty or online (www.dagitty.net)

```
dag {
bb="-1.478,-1.935,2.008,1.677"
"Contact Frequency" [pos="0.285,0.712"]
"Date of Participation" [pos="1.479,-1.770"]
"Household Composition" [pos="-0.476,-1.649"]
"Infection risk" [outcome,pos="1.491,0.247"]
"Migration Status" [adjusted,pos="-0.015,-1.784"]
"Mobility / Mode of Transport" [latent,pos="0.337,0.421"]
"Place of Residence" [adjusted,pos="0.483,1.158"]
"Protective Behaviour" [latent,pos="0.285,-1.065"]
"Risk perception" [latent,pos="0.279,-0.843"]
"Urban vs. Rural dwelling" [adjusted,pos="0.634,1.360"]
"Working from Home" [pos="0.296,-0.030"]
Age [adjusted,pos="-0.953,1.067"]
Education [exposure,pos="-1.024,0.205"]
Employment [pos="-0.457,-1.261"]
Housing [pos="0.285,-0.470"]
Occupation [pos="-0.434,1.184"]
Sex [adjusted,pos="-0.943,-0.960"]
"Contact Frequency" -> "Infection risk"
"Date of Participation" -> "Infection risk"
"Household Composition" -> "Infection risk"
"Household Composition" -> "Working from Home"
"Household Composition" -> Employment
"Migration Status" -> "Date of Participation"
"Migration Status" -> "Infection risk"
"Migration Status" -> Housing
"Migration Status" <-> Education
```

"Mobility / Mode of Transport" -> "Infection risk"

"Place of Residence" -> "Contact Frequency"

"Place of Residence" -> "Date of Participation"

"Place of Residence" -> "Infection risk"

"Place of Residence" -> "Working from Home"

"Place of Residence" <-> Education

"Protective Behaviour" -> "Infection risk"

"Risk perception" -> "Infection risk"

"Risk perception" -> "Protective Behaviour"

"Urban vs. Rural dwelling" -> "Contact Frequency"

"Urban vs. Rural dwelling" -> "Infection risk"

"Urban vs. Rural dwelling" -> "Working from Home"

"Urban vs. Rural dwelling" <-> Education

"Working from Home" -> "Infection risk"

Age -> "Infection risk"

Age -> "Protective Behaviour"

Age -> "Risk perception"

Age -> "Working from Home"

Age -> Education

Age -> Employment

Age -> Housing

Age -> Occupation

Education -> "Contact Frequency"

Education -> "Household Composition"

Education -> "Infection risk" [pos="0.105,0.237"]

Education -> "Mobility / Mode of Transport"

Education -> "Protective Behaviour"

Education -> "Risk perception"

Education -> "Working from Home"

Education -> Employment

```
Education -> Housing

Education -> Occupation

Employment -> "Contact Frequency"

Employment -> "Mobility / Mode of Transport"

Employment -> "Protective Behaviour"

Employment -> "Risk perception"

Employment -> "Working from Home"

Housing -> "Infection risk"

Occupation -> "Contact Frequency"

Occupation -> "Infection risk"

Occupation -> "Mobility / Mode of Transport"

Occupation -> "Working from Home"

Sex -> "Infection risk"

Sex -> "Protective Behaviour"

Sex -> "Risk perception"

Sex -> "Working from Home"

Sex -> Education

Sex -> Employment

Sex -> Occupation

}
```

Supplementary Material 3 Odds ratios (OR) and 95% confidence intervals (95% CI) for SARS-CoV-2 infections by educational attainment, frequency of working from home and the interaction between education (exposure) and frequency of working from home (outcome)

		OR	95% CI	p-value
Educational attainment	Very high (ref.)			
	High	1.66	0.76 – 3.59	0.202
	Medium	1.78	0.87 – 3.63	0.112
	Low	1.63	0.71 – 3.77	0.251
Work from home	No possibility (ref.)			
	≤ Every two weeks	1.56	0.54 – 4-47	0.407
	Several times per week	0.62	0.2 – 1.91	0.403
	Daily	0.70	0.28 – 1.77	0.252
Educational attainment # work from home	Low # ≤ Every two weeks	0.31	0.04 – 2.21	0.231
	Low # Several times per week	empty		
	Low # Daily	empty		
	Medium # ≤ Every two weeks	0.32	0.1 – 1.07	0.063
	Medium # Several times per week	1.29	0.37 – 4.49	0.693
	Medium # Daily	0.75	0.25 – 2.26	0.610
	High # ≤ Every two weeks	0.79	0.22 – 2.84	0.717
	High # Several times per week	1	0.27 – 3.72	0.997
	High # Daily	0.97	0.28 – 3-36	0.370

Chi square test (null hypothesis: Educational attainment # Work from home = 0):

Chi square = 11.12, p-value = 0.677

Supplementary Material 4 Relative risk of SARS-CoV-2 infection by educational attainment and frequency of working from home -Comparison of the estimates from a Model without adjustment for household composition (Model1) and with adjustment for household composition (Model 2)

		**Model 1**	**Model 2**	**Model 1**	**Model 2**	**Model 1**	**Model 2**
		**PR**	**PR**	**PR**	**PR**	**PR**	**PR**
**Education**	**Very high (ref.)**	(ref)	(ref)			(ref.)	(ref.)
	**High**	1.51	1.51			1.34	1.34
	**Medium**	1.66	1.68			1.34	1.36
	**Low**	1.76	1.71			1.27	1.26
**Possibility to work from home**	**No possibility**			(ref.)	(ref.)	(ref.)	(ref.)
	**≤ Every two weeks**			0.85	0.85	0.88	0.88
	**Several times per week**			0.61	0.60	0.65	0.64
	**Daily**			0.55	0.55	0.59	0.60

PR = prevalence ratio

Model 1 = Poisson regression model adjusted for age, sex, migration experience, federal state and urban versus rural residence.

Model 2 = Poisson regression model adjusted for age, sex, migration experience, federal state, urban versus rural residence and household composition.
